# Integrated management of atypical parkinsonism: a home-based patient-centered healthcare delivery based on telenursing—the IMPACT study protocol

**DOI:** 10.1177/17562864241299347

**Published:** 2025-04-03

**Authors:** Roberto Cilia, Fabiana Colucci, Antonio Suppa, Francesca Valentino, Carmen Terranova, Catia Leuzzi, Jessica Cordasco, Giulia Fusi, Simona Floridia, Francesca De Giorgi, Roberta Telese, Arianna Braccia, Alessandro Zampogna, Giulia Pinola, Martina Patera, Giorgio Belluscio, Sara Crivellari, Elisa Antoniazzi, Simona Cascino, Antonio Giaco, Alessio Masaracchio, Giacomina Clara Moreschi, Marisa Catotti, Roberto Eleopra

**Affiliations:** Department of Clinical Neurosciences, Parkinson and Movement Disorders Unit, Fondazione IRCCS Istituto Neurologico Carlo Besta, via Celoria 11, Milano 20133, Italy; Department of Clinical Neurosciences, Parkinson and Movement Disorders Unit, Fondazione IRCCS Istituto Neurologico Carlo Besta, Milano, Italy; Department of Neuroscience and Rehabilitation, University of Ferrara, Ferrara, Italy; Parkinson and Movement Disorders Unit, IRCCS Istituto Neurologico Mediterraneo Neuromed, Pozzilli, Italy; Department of Human Neurosciences, Sapienza University of Rome, Italy; Parkinson’s Disease and Movement Disorders Unit, IRCCS Mondino Foundation, Pavia, Italy; Department of Clinical and Experimental Medicine, University of Messina, Messina, Italy; D.A.P.S. Fondazione IRCCS Istituto Neurologico Carlo Besta, Milano, Italy; Department of Clinical Neurosciences, Parkinson and Movement Disorders Unit, Fondazione IRCCS Istituto Neurologico Carlo Besta, Milano, Italy; Unit of Informative Services, Fondazione IRCCS Istituto Neurologico Carlo Besta, Milan, Italy; Unit of Informative Services, Fondazione IRCCS Istituto Neurologico Carlo Besta, Milan, Italy; Unit of Informative Services, Fondazione IRCCS Istituto Neurologico Carlo Besta, Milan, Italy; Department of Clinical Neurosciences, Parkinson and Movement Disorders Unit, Fondazione IRCCS Istituto Neurologico Carlo Besta, Milano, Italy; Department of Clinical Neurosciences, Parkinson and Movement Disorders Unit, Fondazione IRCCS Istituto Neurologico Carlo Besta, Milano, Italy; Parkinson and Movement Disorders Unit, IRCCS Istituto Neurologico Mediterraneo Neuromed, Pozzilli, Italy; Department of Human Neurosciences, Sapienza University of Rome, Italy; Department of Human Neurosciences, Sapienza University of Rome, Italy; Department of Human Neurosciences, Sapienza University of Rome, Italy; Parkinson’s Disease and Movement Disorders Unit, IRCCS Mondino Foundation, Pavia, Italy; Parkinson’s Disease and Movement Disorders Unit, IRCCS Mondino Foundation, Pavia, Italy; Parkinson’s Disease and Movement Disorders Unit, IRCCS Mondino Foundation, Pavia, Italy; Department of Clinical and Experimental Medicine, University of Messina, Messina, Italy; Department of Clinical and Experimental Medicine, University of Messina, Messina, Italy; Department of Clinical and Experimental Medicine, University of Messina, Messina, Italy; D.A.P.S. Fondazione IRCCS Istituto Neurologico Carlo Besta, Milano, Italy; D.A.P.S. Fondazione IRCCS Istituto Neurologico Carlo Besta, Milano, Italy; Department of Clinical Neurosciences, Parkinson and Movement Disorders Unit, Fondazione IRCCS Istituto Neurologico Carlo Besta, Milano, Italy

**Keywords:** atypical parkinsonism, case manager, healthcare, MSA, Multidisciplinary, PSP, telemedicine, telenursing, wearable

## Abstract

**Background:**

People with atypical parkinsonism, such as multiple system atrophy and progressive supranuclear palsy, experience a wide range of motor and non-motor symptoms associated with the increasing complexity of care delivery and the increased risk of complications and hospital admissions.

**Objectives:**

To investigate the efficacy and cost-effectiveness of a 12-month remote home-based integrated program aiming to improve healthcare delivery coordinated by a nurse specialized in the management of individuals with atypical parkinsonism (parkinsonism nurse specialist, PKNS) compared to the standard-of-care model.

**Design:**

Multicenter, randomized, single-blind, controlled clinical trial involving 164 individuals with atypical parkinsonism.

**Methods and analysis:**

Participants will be randomized 1:1 in intervention (PKNS) and control (standard-of-care) arms. Assessments will be undertaken at baseline and after 6 and 12 months. Primary outcome measure is the Parkinson’s Disease Questionnaire 39-items scale total score. Secondary measures include the clinical scales testing motor and non-motor symptoms, caregiver burden, adherence to therapy, cumulative disease burden and the number of unplanned hospital visits/admissions during the study period. The cost-effectiveness of this method will be evaluated by using the EuroQoL-5, which estimates the incremental cost per quality-adjusted life-years gain. Real-life motor autonomy will be objectively measured by collecting waist-worn wearable data on gait parameters (automatically detecting motor patterns indicative of freezing of gait and falls) in all subjects for five consecutive days each month during the 12-month duration of the study.

**Ethic:**

Study protocol has been approved by the ethics committee of all participating centers. The study is conducted according to good clinical practice and the Declaration of Helsinki.

**Discussion:**

An integrated remote care model at home coordinated by a specialized nurse in the management of parkinsonism (Telenursing) could offer significant benefits to patients and healthcare professionals through better health education, continuity of care, and careful monitoring of complications.

**Trial registration:**

ClinicalTrials.gov identifier NCT05792332.

## Background

Parkinsonism (PKS) refers to a clinical syndrome characterized by bradykinesia, rigidity, gait, and postural disturbances. People living with parkinsonism (PwP) experience a wide range of motor and non-motor symptoms and high complexity in care management, inevitably leading to significantly higher disability and burden on healthcare resources. Therapeutic intervention in PwP goes beyond pharmacological therapy by integrating a wide variety of interventions typical of nursing management, and it aims at ensuring greater control of motor symptoms (e.g., prevention of falls and bone fractures) and non-motor symptoms (e.g., dysphagia, orthostatic hypotension, constipation, micturition disorders, sleep disorders, etc.) and the maintenance of functional autonomy. These are two fundamental prerequisites to achieve the goal of adequate management of chronic neurodegenerative diseases.

An integrated approach, involving a nurse specialized in parkinsonism (parkinsonism nurse specialist, PKNS) alongside a neurologist could offer significant benefits to patients and care providers through improved health education, continuity of care, and prevention and close monitoring of incident complications.^1,2^ Several studies highlight how a multidisciplinary approach including a PKNS can offer significant benefits to patients and care providers in the management of disability caused by motor and non-motor symptoms and in monitoring side effects and adherence to therapy.^3,4^ A PKNS may be the most suitable candidate to the “first point of access” for queries raised by patients and/or care partners and create a filter for the different assistance requests through a triage and dedicated referral.^2^ In a hub-and-spoke model, the PKNS may be the joining link between a tertiary referral center (“hub”) and nearby community hospitals (“spoke”). PKNS can play a fundamental role in shifting and sharing tasks in the management of people with PKS, significantly contributing to alleviating the care burden on neurologists. In this scenario, the PKNS may be defined as “case manager” who can filter assistance requests and independently manage many motor and non-motor problems to involve the neurologist only if necessary. In several regions such as the UK,^5,6^ nurses are granted the authority to prescribe medicines and play a vital role in maintaining continuity of care for chronic diseases by coordinating care between patients and primary and secondary care providers.^7^ Guidelines for PD nurse specialists have been developed in UK, the Netherlands, and Germany,^1,3,8^ whereas is Italy the value and the authority of the PD nurse specialist is still under-recognized and we feel that a cultural evolution is urgently needed.

In the absence of specialized support, patients and/or care partners may either underestimate symptoms that require immediate therapeutic intervention or overestimate them and increase utilization of healthcare resources by unnecessary emergency room visits and consultations and greater number of hospitalizations. Falls, urinary infections, and dementia account for 24%, 16%, and 13% of emergency room access costs, respectively.^9^ Disease progression leads to increased risks of morbidity and subsequent mortality from complications due to motor disability (e.g., falls, fractures, dysphagia, infections) and non-motor disability (e.g., dementia, psychosis, dysautonomia). When patients with parkinsonism are hospitalized, the length of stay increases,^10^ the risk of infections, prescribing errors, increased postoperative mortality^11,12^ with increased risk of re-hospitalization.^13^ A study in the United States reported a significant economic burden due to hospitalization of patients with parkinsonism: about 20% of the estimated annual cost per patient ($4.6 billion US) was due to hospital costs.^10^ Several retrospective studies have shown that frequent neurological consultations and adherence to the drug therapy schedule can reduce hospitalization by up to 50%.^12^ Falls, fractures, infections, and cognitive and motor decline have been identified as risk factors for unplanned hospitalizations in patients with parkinsonism.^12,13^ These complications are more frequent in primary atypical parkinsonism such as multiple system atrophy (MSA) and progressive supranuclear palsy (PSP) than in PD, because of the greater extent and rapidity of the neurodegenerative process, the reduced efficacy of drug therapy or more common drug-related side effects. Given the magnitude of hospitalizations in PwP, associated morbidity, mortality, and healthcare-related costs, there is a need for cost-effective interventions to reduce unplanned hospitalization by acting on preventable risk factors.^14^ Optimizing clinical management of motor and non-motor symptoms and pharmacological side effects, through a telenursing service may prevent falls and hospital admissions, with an increased quality of life and a reduction of comorbidity and risk of caregiver’s burnout.

## Rationale

The aim of the IMPACT project is to measure the effectiveness of a specialized healthcare model governed by a case manager via telemedicine dedicated to coordinating the care activity offered to patients with atypical parkinsonism in comparison with the standard-of-care model in terms of improving the quality of life. To date, no study has ever reported the effectiveness of a telemedicine service managed by a PKNS who take care of the patient by telephone and follow an Integrated Care Plan (herewith defined as “Telenursing”).

The IMPACT project is designed to investigate the benefits and cost-effectiveness of an integrated patient-centered care to propose this model to stakeholders as a new pragmatic organizational model to foster continuity of care by exploiting the potential of existing care resources. This model implements a personalized telenursing-based approach with the patient at home, including through a passive telemonitoring system of the patient’s mobility and risk of falls. As an alternative to the usual clinical model of care, in this model people with parkinsonism are proactively followed remotely by a PKNS, who can prevent and minimize complications caused by the disease and/or medication side effects (e.g., falls and fractures caused by instability or orthostatic hypotension) by promoting flexible, personalized, and comprehensive care. It is expected that this telenursing-based model will simplify care delivery by reducing the incidence of complications and unplanned hospital admissions.

Many elements of the IMPACT innovative and timely design can be widely implemented in different healthcare settings and become part of our new way of managing patients with chronic neurological diseases, including but not limited to patients with rare, atypical parkinsonism. We will use a semi-structured flowchart that the case manager follows (1) to create a “tailor-made” file of the patient on entry to the practice, (2) proactively follow up on the most relevant motor and non-motor problems to prevent complications, and (3) to use a flowchart predefined by nurses to organize incoming calls (reactive action) and define the triage code to alert the neurologist and general practitioner for activation of the multidisciplinary team. The application of new wearable sensors and advanced machine learning algorithms will objectively monitor the real-life autonomy motor in a home environment and further indicate the innovative aspect of our research project.^15^ Efficacy will be assessed both by estimating the improvement in quality of life and coping mechanisms related to motor and non-motor disorders and by weighing the reduction in any unplanned hospital admissions (e.g., requests for unplanned “extra” neurological visits, any hospital or emergency room admission).

## Methods and analysis

### Study design

Multicenter case-control, nonprofit, randomized 1:1, single-blind, parallel-group clinical trial to recruit 164 patients (*n* = 82 per arm) with atypical parkinsonism (MSA or PSP) from the four operating units involved. The study duration per single patient will be 12 months. The study sites include Fondazione IRCCS Istituto Neurologico Carlo Besta (Milan), IRCCS Mondino Foundation (Pavia), IRCCS Istituto Neurologico Mediterraneo Neuromed (Pozzilli), and Gaetano Martino University Hospital (Messina).

The study has been approved by each centers Ethics Board (Protocol ID: IMPACT). The trial has been registered on ClinicalTrials.gov (ID: NCT05792332).

### Recruitment and participants

Participants must fulfill the following inclusion criteria: (1) age 40–85 years and (2) clinical diagnosis of MSA^16^ or PSP^17^ in all their possible variants (MSA of parkinsonian vs cerebellar type;^16^ PSP Richardson Syndrome, PSP-Parkinsonism, PSP-Corticobasal Syndrome, etc.^17^) according to internationally validated criteria. Exclusion criteria include: (1) Hoehn and Yahr stage 5 in ON phase (bedridden patients); (2) Clinical Frailty Scale (CFS) ⩾ 8^18^; (3) dementia according to Diagnostic and Statistical Manual of Mental Disorders 5th edition (DSM-V) criteria; and (4) serious medical disorders that, in the opinion of the recruiting neurologist, may impair participation in the study.

Potential participants will be recruited at each center. Individuals referred to the study will be pre-screened via chart inspection and offered a screening visit if they meet inclusion criteria. At the screening visit, the inclusion and exclusion criteria will be reviewed. Participants are consecutively enrolled by the investigating neurologist of the participating centers after obtaining and signing an informed consent form. Forms for registering patients evaluated for possible study participation and those enrolled must be completed and kept by each experimental center (with a copy to the sponsor). A form for identification of all patients (with study code and patient identification data) who were screened for the study should be completed and filed at the center (Subject Identification Code List).

Then, participants’ demographic information, medical history, and concomitant medication list will be recorded. Contacts and minimum clinical data useful for stratification (see below) will be addressed to the Clinical Research Coordinator (CRC) of the coordinating center.

### Randomization and blinding

Eligible patients will be randomized with 1:1 allocation into the two treatment arms with a centralized web-based randomization system. The randomization list will be generated by computer. Randomization is stratified by (1) sex, (2) diagnosis (MSA vs PSP), and (3) disability (CFS ⩽ 4 vs. > 4). For blinding purposes, the CRC schedules evaluation by a blind neurologist (“blind rater”) of all enrolled patients, blind to randomization. The blind rater will perform the scales and questionnaires prescribed in the protocol. The patient knows if he/she will follow the “case-manager” procedure or if he/she will be approached with the standard-of-care model.

### Intervention and monitoring

#### Intervention arm

Patients in the intervention arm will receive care from a PKNS for any medical (non-administrative) needs tailored to their necessities and standard medical treatment. A PKNS from each site will follow the patients enrolled.

- *PROACTIVE monitoring*: At baseline (V1), the PKNS contacts the patient to create a personal record using a semi-structured interview, including all symptoms of motor and non-motor characteristics and the type of risks that may occur. The PKNS instructs on risk prevention strategies related to the most relevant issues reported. After the initial proactive call to V1, PKNS will provide intermediate contacts every 3 months. At intermediate calls (V2, V3, V4), the PKNS focuses the interview on the problems/risks detected at baseline and provides tailored counseling and education. At the final visit (V5), the investigator will complete the follow-up of all the incident events and medical issues occurred during the study.
- *REACTIVE monitoring*: During the study period, the patient and care provider may contact the PKNS at any time prior to the previously scheduled in-person visit for urgent needs by telephone (3h/day, 5 days/week, Mon-Fri) or by e-mail (within 48 h).

The PKNS can (1) also address the specific problem by interacting with the participant’s primary care physician and other healthcare providers or (2) refer to the treating neurologist (who is not the blind rater) for teleconsultation or in-person visit, according to a semi-structured algorithm that assigns four levels of priority similar to emergency room “triage”.^19^ Briefly, whenever a referral to a neurologist is needed, PKNS assigns a 4-level code triage, where increased urgency would lead to reduced assessment time and the opportunity for a teleconsultation with an experienced neurologist. We proposed this approach to triage patients query during COVID-19 pandemic, including a detailed flowchart.^19^ The code colors are summarized in Table 1. In this project, we have elaborated the “*case manager triage*” process by creating a semi-structured interview based on (1) the timing of the new event, and (2) the type of motor or non-motor issue. The code assignment is based on two step questions: (a) Does this change increase the risk of short-term morbidity (e.g., risk of hip fracture due to falls)? (b) Has functional independence changed? According to the answers to questions “a” and “b”, there will be four scenarios: (1) code white (answer: no/no): non-urgent, resolvable via e-mail/phone; (2) green code (answer: no/yes): not urgent but requires teleconsultation; (3) code yellow (answer: yes to “a”): timely teleconsultation to resolve urgent matters and decide whether an in-person visit is necessary; and (4) code red: need for hospitalization. The total scores suggest the code assignment (Table 2).

**Table 1.** Triage performed by the nurse specialist when the patient query needs to be addressed by the neurologist.

Triage code^a^	Severity	Action	Aim	Timing
White	Non-urgent	E-mail or Telephone call	Solve the query	3 working days
Green	Non-urgent	Teleconsultation	Solve the query	10 working days
Yellow	Urgent	Teleconsultation	Solve the query Decide if in-person visit is needed	3 working days
Red	Urgent	Immediate telephone call Schedule in-person visit	Urgently solve the query	1 working day

a The code assignment is based on two step questions: (a) Does this change increase the risk of short-term morbidity? (b) Has functional independence changed? See the manuscript (page 7, lines 16–22) for details and Table 2 for the scoring system.

**Table 2.** Triage code color assignment based on the timing and severity of motor and non-motor issue.

Timing/Frequency	Scoring	Partial score
Timing of event		
<7 days	3	
7–30 days	2	
>30 days	1	
None	0	
Falls		
Every day	4	
1–2 times/week	2	
1–2 times/month	1	
None	0	
Dysphagia		
Almost every meal	4	
Once/week	2	
Occasional	1	
None	0	
Constipation		
<1 evacuation/week	4	
2–3 evacuation/week	2	
Occasional issue	1	
None	0	
Orthostatic Hypotension		
Every day	4	
1–2 times/week	2	
1–2 times/month	1	
None	0	
Nocturia		
⩾3 times/night	4	
1–2 times/night	2	
Occasional	1	
None	0	
Pain		
Every day	4	
1–2 times/week	2	
1–2 times/month	1	
None	0	
Agitation/Psychosis		
Continuous (day and night)	4	
Every day, only certain times, manageable by caregiver	3	
Every day, only certain times, not manageable by caregiver	2	
Occasional	1	
None	0	
Total score		
Code		
White		⩽2
Green		3–6
Yellow		7–12
Red		⩾13

#### Control arm

Patients in the control arm will undergo a 30-min in-person visit with the neurologist at baseline (V1), 6 months (V3), and 12 months (V5) after randomization.

### Procedures

#### Clinical assessment

After the screening visit (eligibility verification according to inclusion and exclusion criteria, informed consent collection, demographic data collection, and clinical data collection, including the CFS), in-person assessment with the blind rater will be performed at baseline (V1) and after 6 months (V3) and 12 months (V5), regardless of allocation arm. All study visits are conducted by neurologists experienced in movement disorders.

**Figure 1.**
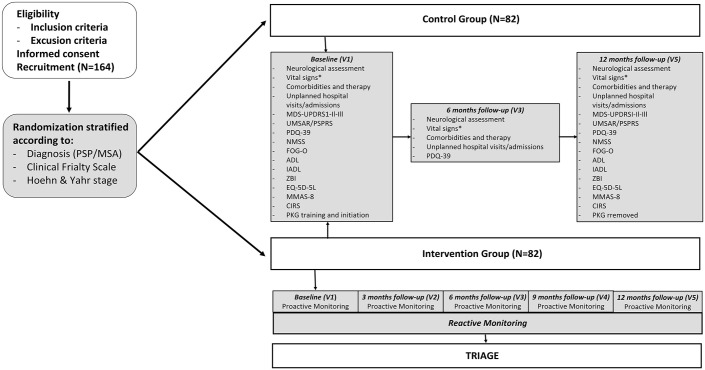
Study design. *Blood Pressure (BP) and Heart Rate (HR) are measured in the supine position and after 3 min of active standing. ADL, Activities of Daily Living; CFS, Clinical Frailty Scale; CIRS, Cumulative Disease Rating Scale for Geriatrics; EQ-5D-5L, European Quality of Life 5 dimensions; FOG-Q, Freezing of Gait Questionnaire; H&Y, Hoehn and Yahr; IADL, Instrumental Activities of Daily Living; MDS-UPDRS, Movement Disorder Society-sponsored revision of the Unified Parkinson’s Disease Rating Scale; MMAS, Morisky Medical Adherence scale-8 items; MSA, Multiple System Atrophy; NMSS, Non-Motor Symptoms Scale; PDQ-39, Parkinson’s Disease Questionnaire-39; PSP, Progressive Supranuclear Palsy; PSPRS, Progressive Supranuclear Palsy Rating Scale; UMSAR, Unified Multiple System Atrophy Rating Scale; ZBI, Zarit Burden Interview scale.

The study flowchart is summarized in Figure 1.

At baseline (V1) and 12 months (V5), the blind rater will collect: demographic and social information (including caregiver’s relationship with the patient (e.g., spouses, siblings, hired caregivers, etc.) and the number of caregivers per patient), general and neurological examination; PD Questionnaire-39 (PDQ-39);^20^ Movement Disorder Society-sponsored revision of the Unified PD Rating Scale (MDS-UPDRS);^21^ Hoehn and Yahr stage (H&Y); Freezing of Gait Questionnaire (FOG-Q);^22^ Unified MSA Rating Scale (UMSARS) in patients diagnosed with MSA or PSP Rating Scale (PSPRS) in patients diagnosed with PSP;^23,24^ Non-Motor Symptoms Scale (NMSS);^25^ Activities of Daily Living (ADL, IADL);^26,27^ Zarit Burden Interview scale (ZBI);^28^ European Quality of Life 5 dimensions (EuroQoL);^29^ Cumulative Disease Rating Scale for Geriatrics;^30^ Morisky Medical Adherence scale-8 items (MMAS-8).^31^ All patients and care providers will be trained on using the wearable sensor and its free downloadable app on their smartphones at the screening assessment and, to confirm understanding, at the baseline visit. At follow-up after 6 months (V3), the blind rater will perform a neurological examination, record any incident medical events (including falls) and hospitalizations, and collect PDQ-39.

#### Objective monitoring of motor function

To achieve objective and quantitative measures of patients’ motor function, we will perform a long-term monitoring of gait through continuous gait monitoring, we will use a single wearable sensor (STAT-ON^®^) SENSE4CARE SL., Cornellà de Llobregat (Spain) on a waist. STAT-ON is a Class IIa inertial medical device that is both lightweight and compact (86 g; 9 × 6.3 × 2.1 cm).^32^ It has successfully passed electromedical equipment tests, including those for home use. The device features two ultra-low triaxial nano-accelerometers, two microcontrollers, and a Bluetooth low-energy system, offering a battery life of 7 days under normal conditions. STAT-ON^®^ provides standard spatiotemporal gait parameters and uses advanced machine-learning algorithms to automatically detect motor patterns indicative of freezing of gait (FOG) and falls.^33^

In this study, following the sensor configuration based on individual clinical data (e.g., age, leg length), all patients will wear the STAT-ON sensor on the left waist via an elastic belt for five consecutive days (for 8 h per day during waking hours) per month. Recording sessions will be acquired monthly for a period of 12 months to collect the following spatiotemporal gait parameters and incident events: (1) number of steps; (2) step length; (3) step velocity; (4) step cadence; (5) number of FOG episodes; and (6) number of falls.^34^ Every 3 months, an encrypted and anonymized version of all collected data will be automatically sent to the PKNS of each center. The collected data will be summarized in a special report, including a graphical representation of the mean trend of parameters and spatiotemporal events of gait over time, as well as quantitative measures. In the intervention arm, physical activity levels and real-life motor autonomy will be objectively assessed to support proactive monitoring by the PKNS, who will add this objective information to the patient’s subjective report to adjust counseling and education during proactive calls made every 3 months. In the control group, data will be collected as a secondary outcome measure for final statistical analysis. At the end of the study, the data obtained from the wearable devices will be used to perform between-group analysis (comparison of cases vs controls) and within-group analysis (changes between V1 and V5 in each study group).

### Outcome measurements

#### Primary outcome

The primary aim is to demonstrate that an integrated care delivery model coordinated by a PKNS results in more favorable changes in quality of life at 12 months than standard medical care. The outcome measure will be the change from baseline to 12-month result in PDQ-39 total score, comparing cases to controls. PDQ-39 scale is a comprehensive assessment battery composed of 39 items grouped into eight subscales (mobility, activities of daily living, emotional well-being, stigma, social support, Cognition, Communication, and Bodily discomfort). Each item scores from 0 (never) to 4 (always); subscale scores and a summary index representing the global health-related quality of life can be obtained. PDQ-39 does not present any relevant ceiling or floor effect and has been demonstrated to be consistent, reliable, and discriminative for disease severity.^35^

#### Secondary outcomes

1. To evaluate the feasibility and effectiveness of an innovative telenursing approach consisting of proactive and reactive remote monitoring aimed at optimizing continuity of care by improving motor and non-motor disability control, including through better adherence (compliance) to the prescribing regimen.
 Outcome measures will be the changes in MDS-UPDRS (Parts I, II, and III) and NMSS scores in all participants. Disease-specific scales will also be used: (a) UMSARS in subjects diagnosed with MSA and (b) PSPRS in those diagnosed with PSP. In addition, changes in scores on the functional disability (CFS) and autonomy in activities of daily living (IADL, ADL) scales will be used. Compliance to prescribing regimen will be measured by the changes in scores on the Italian version of the MMAS-8 on adherence to pharmacological therapy.^31^
2. To investigate the cost-effectiveness of the management model by telenursing compared to standard care in terms of (a) reducing the number of unplanned hospital admissions and (b) reducing complications/comorbidities to minimize direct and indirect costs to the national healthcare system.
 We will analyze differences between the two study groups in the number of unplanned hospital admissions in 12 months through an interview to patient and/or care provider (calculating outpatient visits, emergency room admissions, hospitalizations directly or indirectly associated with neurological pathology). In addition, differences between the two study groups in comorbidity frequency (total events, disease-related events) through (a) patient and/or caregiver interview and (b) the Italian version of the Cumulative Illness Rating Scale for Geriatrics^30^ will be evaluated. To estimate the incremental cost per quality-adjusted life-year gain (QALY)^29^ change from baseline to 12 months of the EuroQoL scale total score will be analyzed.
3. To evaluate the feasibility and utility of telemonitoring to improve PKNS and neurologist decision-making using a device worn at the waist for 5 days per month (8 h/day), which passively collects data on real-life motor autonomy and fall risk, including FOG episodes.
 Changes in gait parameters in both between-group (between-group) and same-patient (within-group) analyses will be evaluated to obtain information on changes in physical activity, motor autonomy, gait disorders (including FOG), and risk of falls.
4. To reduce the risk of caregiver burnout. Changes in the ZBI on caregiver burnout^28^ from baseline to 12-month follow-up will be used.
5. Patient satisfaction by evaluating the differences to the patient satisfaction questionnaire.

### Safety

All adverse events (AEs) including all Serious adverse events will be collected, properly investigated, and documented in the original documents and appropriate sections of the eCRF during the entire study period, from the signing of informed consent to the last protocol-specific procedure, including the follow-up period for safety.

### Analysis plan

#### Simple size

This is the first study to investigate the efficacy of an integrated program in patients with rare forms of parkinsonism such as MSA and PSP, whereas this has been previously studied only in PD.^4^ Considering that there is no validated questionnaire measuring quality of life in MSA and/or PSP, we relied on data from a previous randomized trial on the effectiveness in terms of health-related quality of life of an integrated program coordinated by a case manager versus the standard of care in PD.^4^ We hypothesized a difference of at least 3, 9 in change at 12 months in the PDQ-39 between the proposed integrated care model and the standard of care, with expected standard deviations of 8.6 in the control group and 5.8 in the treated group. Considering a type I error of the two-tailed *t*-test of 5%, a power of 90% and a loss to follow-up of no more than 10%, a sample size of 164 patients (82 per arm) is needed. Although patients with MSA and PSP are expected to show a more aggressive disease course than PD with a more rapidly worsening course of disability and quality of life than idiopathic PD, we decided to maintain a similar effect size to compensate for the potential lower specificity of the primary outcome measure (e.g., PDQ-39).

#### Statistical analysis

No intermediate analysis is planned. The primary analysis will be by intention to treat principle as far as is practically possible. Analysis by protocol (PP) will be done after excluding patients with major deviations from the protocol. Descriptive statistics by treatment arm will be provided for all variables assessed in the study protocol with mean and standard deviation and median with range for continuous variables and absolute numbers and percentages for categorical variables.

The primary analysis will be based on the two-tailed *t*-test. Changes from baseline in the primary and secondary continuous variables (including longitudinal data obtained from the wearable device) will be analyzed by analysis of variance for repeated measures adjusted for baseline, or a corresponding nonparametric analysis as appropriate. Safety analysis will be performed by calculating the proportion of patients with AEs. The *p* values < 0.05 will be considered statistically significant, and all tests will be two-sided. The STATA Statistical Software, Release 16 (StataCorp. 2019. Stata Statistical Software: Release 16. College Station, TX: StataCorp LLC) will be used for statistical analysis.

### Data management and monitoring

The Clinical Research Center of coordinating center (Fondazione IRCCS Istituto Neurologico Carlo Besta) will generate codes for randomization and assignment to study arms. To preserve anonymity, the patient will be identified by an alphanumeric code.

The centers will collect all demographic and clinical data using codes; the data and database will be password-protected, with access restricted to authorized study personnel only. Data will be collected in real-time for each individual survey through an electronic acquisition system by filling out an electronic Data Collection Form (Case Report Form, eCRF) created with the Ticuro^®^, Italy software currently in use at the coordinating center, access to which will be granted to partner centers through credentials provided only to investigators in charge of individual centers and authorized study personnel (in delegation). The Ticuro platform ensures the security and confidentiality of clinical information. All sensitive data are encrypted and protected, following current regulations on health data privacy and security (ISO 13485, ISO 9001, ISO 14001, MDR CE), allowing access only to authorized healthcare professionals. In addition, thanks to special auditing mechanisms, Ticuro ensures traceability of decisions made during the care/trial process and efficient management of clinical information.

Among the certified modules of the Ticuro Platform is the Management of Patient-Reported Experience Measures and Patient-Reported Outcome Measures module, which enables the creation, delivery, and historicization of digitized questionnaires. This functionality generates eCRFs (Electronic Case Report Forms) that can be combined to generate clinical case presentations for scientific publication. This module also enables data analysis in full compliance with the above rules.

The completeness and accuracy of the compilation of the data transcribed on the eCRF will be checked by verifying its correct execution from the compilation history on the platform. For analysis, the data will be extracted from the e-CRFs into a document in json format, keeping the identification of the subjects a pseudonymized code (in accordance with the GDPR). The Principal Investigator ensures that the study is conducted in accordance with this protocol, Good Clinical Practice, the current version of the Declaration of Helsinki, and applicable regulations.

All patients will be informed of the objectives of the study. General practitioners will be informed of the strict confidentiality of their patient’s data, but their medical records may be reviewed for study purposes by authorized persons other than their treating neurologist. When data entry is complete (database “completed”), data quality checks will then be performed. The database will then be corrected. When corrections are completed (database “cleaned”), the database will be frozen (database “closed”). Correction of data after the database is closed must be approved by the Research Manager and must be properly documented.

### Data collection

The investigator will retain data and documents related to the conduct of this study according to current legal regulations. After this period, the documents may be destroyed according to local regulations.

## Discussion

Quality of life is the cardinal objective of atypical parkinsonism patients care. Severe complications associated with the numerous comorbidities typical of aged frail patient characterize the natural history of atypical parkinsonism.

The present trial is the first to test the effectiveness of a telemedicine service run by movement disorder nurses who take charge of patients with atypical parkinsonism following an Integrated Care Plan. The trial will include particularly frail patients with Parkinsonism, and efficacy will be assessed by calculating the improvement in quality of life and coping mechanisms related to motor and non-motor disorders and reduction in unplanned hospital admissions. Moreover, an ad hoc qualitative questionnaire has been designed to assess patient and caregiver perception and satisfaction with the quality of healthcare provided. Similar questionnaires had been used in previous clinical trials.^36–38^

Unquestionably, the development of a patient-centered care model is one of the greatest future challenges for public national healthcare systems, preventing their collapse under the increasing costs of disability caused by degenerative disorders.

This practice is associated with several benefits not only for the patient but also for public health, clinical practice. The presence of a specialized nurse at the center of a healthcare model, acting as an intermediary between patient/caregiver, neurologist, general practitioner, and a multidisciplinary team, should promote a more efficient decision-making process and thus more efficient problem-solving, ultimately improving the quality of life of the patient and caregiver. The study population is frail patients with highly disabling chronic diseases that have a high impact on the NHS in terms of direct and indirect costs, and our model is expected to improve public health by reducing complications and unexpected hospital access. This telemedicine-based approach can also benefit patients living in rural areas or far from specialized centers.

This paradigm has the potential to become a new standard of care transferable to patients with other neurological disorders and conditions associated with non-neurological frailty.

This new nurse-based model of care and management of patients with rare, chronic, and disabling diseases with high-risk social burden, including caregiver, can be extended to other more common chronic diseases and throughout the territory with a hub-and-spoke model, where third-level centers with specialized nurses handle more complex cases and provide training to community nurses. An important implication is the potential reduction in economic burden on the NHS in terms of numbers of unplanned hospital admissions for complications of falls from instability and/or hypotension with fractures, *ab ingestis* pneumonia due to dysphagia (early and common in both MSA and PSP).

There are limitations to acknowledge. First, this study focuses on atypical parkinsonisms such as MSA and PSP. These findings will need to be replicated in larger cohorts of patients with Parkinson’s disease and also in patients with dementia associated with parkinsonism. Second, this telemedicine-based approach may be limited to telephone calls by the neurologist (rather than teleconsultation, see Table 1) in patients with limited access or limited ability to use Internet, such as those living in rural areas and/or the elderly or cognitively impaired individuals without a caregiver.

### Study state

Recruitment started on August 11th 2023, and it is planned to be completed by November 17th 2024. Recruitment and the trial status are active and ongoing.

## Declarations

**Ethics approval and consent to participate:** The present study has been approved by Ethics Board of each center (Protocol ID: IMPACT), and all individuals signed a consent form before participation. The EC of the coordinating center approved the study on February 8th, 2023. The study coordinator reviewed the letter of information, answered questions, and obtained consent from the participant and/or caregiver at the screening visit. Because the ability to consent could be difficult to assess and could change over time, written consent was obtained from both participants with dementia and caregiver/next of kin to ensure the rights of participants with deficits in comprehension or decision-making.

**Consent for publication:** Not applicable.

**Availability of data and materials:** Not applicable.
